# ClothFace: A Batteryless RFID-Based Textile Platform for Handwriting Recognition

**DOI:** 10.3390/s20174878

**Published:** 2020-08-28

**Authors:** Han He, Xiaochen Chen, Adnan Mehmood, Leevi Raivio, Heikki Huttunen, Pasi Raumonen, Johanna Virkki

**Affiliations:** 1 Faculty of Medicine and Health Technology, Tampere University, 33720 Tampere, Finland; xiaochen.chen@tuni.fi (X.C.); adnan.mehmood@tuni.fi (A.M.); johanna.virkki@tuni.fi (J.V.); 2 Faculty of Information Technology and Commication Sciences, Tampere University, 33720 Tampere, Finland; leevi.raivio@tuni.fi (L.R.); heikki.huttunen@tuni.fi (H.H.); pasi.raumonen@tuni.fi (P.R.)

**Keywords:** human–machine interaction, passive UHF RFID, textile electronics, user interface, wearables, deep learning

## Abstract

This paper introduces a prototype of ClothFace technology, a battery-free textile-based handwriting recognition platform that includes an e-textile antenna and a 10 × 10 array of radio frequency identification (RFID) integrated circuits (ICs), each with a unique ID. Touching the textile platform surface creates an electrical connection from specific ICs to the antenna, which enables the connected ICs to be read with an external UHF (ultra-haigh frequency) RFID reader. In this paper, the platform is demonstrated to recognize handwritten numbers 0–9. The raw data collected by the platform are a sequence of IDs from the touched ICs. The system converts the data into bitmaps and their details are increased by interpolating between neighboring samples using the sequential information of IDs. These images of digits written on the platform can be classified, with enough accuracy for practical use, by deep learning. The recognition system was trained and tested with samples from six volunteers using the platform. The real-time number recognition ability of the ClothFace technology is demonstrated to work successfully with a very low error rate. The overall recognition accuracy of the platform is 94.6% and the accuracy for each digit is between 91.1% and 98.3%. As the solution is fully passive and gets all the needed energy from the external RFID reader, it enables a maintenance-free and cost-effective user interface that can be integrated into clothing and into textiles around us.

## 1. Introduction

Human–machine interaction is used for inputting and outputting information through touch or human body movement. The most common on-body interfaces such as touchpads and tapping buttons [1,2,3,4], are usually integrated around the arm for detecting hand movement. Recently, versatile technologies have been suggested for on-body touch and gesture recognition, such as skin electronics [5], different types of sensors [4,5,6] and interactive textiles [7,8]. However, these solutions usually require complex electronics and/or on-board power sources, which limit the flexibility, scalability, cost and working life in a daily use.

In addition, vision-based methods have been utilized to capture touch traces on surfaces by using multiple cameras [9,10,11]. However, a line-of-sight is needed in this type of solution, which significantly limits the usability and mobility of these systems. To overcome these challenges, gesture monitoring without wearable devices has been suggested by using Wi-Fi signals [12,13]. However, these solutions have challenges with multiple user environments. Furthermore, they are configuration-dependent and only useful in a specific use environment [14].

The properties of passive ultra-high frequency (UHF) radio frequency identification (RFID) technology make it an attractive solution for human–machine interfaces [15,16,17]. This technology is passive and maintenance-free, does not require a line-of-sight and has reading distances of several meters (even through materials). Prior works have primarily relied on RSSI (Received Signal Strength) [16,18] or AoA (Angle of Arrival) information [19]. When a person touches or moves an RFID tag, it manifests as a traceable change in the backscattered signal, which can be turned into an input to digital actions. However, many factors in the practical use environments, such as electronic devices, wooden and metallic furniture, as well as human bodies, cause the backscattered signals of RFID tags to be noisy and unstable.

Recently, RFID-based touchpads utilizing on/off performance of the ICs (integrated circuits) were studied to track expressive gesture inputs on a platform [20,21]. In this method, touching the pads will create an electrical connection that allows reading of the IDs of the touched ICs. Thus, instead of changes in the backscattered signal properties, IDs of the ICs are tracked. Thus, the environmental disturbances are minimized. However, these platforms were not fabricated on a textile, thus they cannot integrate into daily clothes. In addition, only simple inputs were recognized in these studies, such as swiping and single touch.

This work is an extended study of our previous work [22], which presented a preliminary prototype of ClothFace technology, a batteryless textile-based handwriting platform. The platform comprises of an e-textile antenna and a 10 × 10 array of UHF RFID ICs, each with a unique ID. In this paper, we further optimize the design of the platform, implement a real-time recognition system and evaluate the practical usability through sufficient user testing. Compared to previous works around the topic [20,21,22], this study provides a fully textile-based batteryless solution that has the possibility to be integrated into everyday clothing. In addition, by taking advantage of machine learning methods, our platform can perform significantly more complex functions. As a result, the platform can recognize handwritten numbers 0–9 in real time with a high accuracy, regardless of the writing habit and usage experience of the user.

## 2. Platform Design and Manufacturing

**Figure 1:**
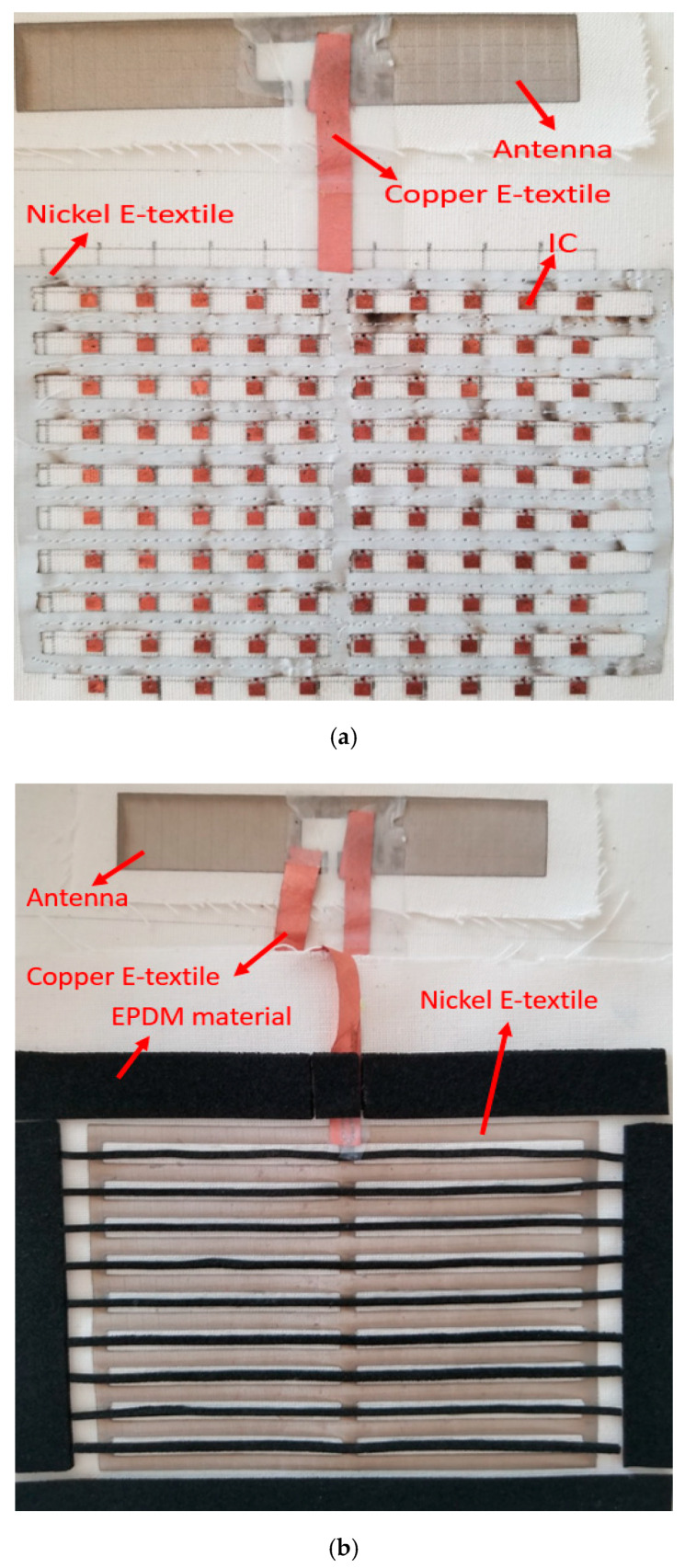
Fabricated platform. (**a**) bottom; (**b**) top layer with separation material.

The textile platform consists of three parts: a bottom layer, a separation layer and a top layer, which is shown in Figure 1. As shown in Figure 1a, the bottom layer is created on a thin cotton-based fabric, with a 10 × 10 array of RFID ICs (NXP UCODE G2iL from NXP Semiconductors, Eindhoven, The Netherlands). The ICs are attached by the manufacturer on a plastic strap with two copper pad endpoints. These straps are fixed on a cotton substrate using a textile glue. The distance between each IC component is 1 cm. On the bottom layer, the right-hand-side copper endpoints of the ICs are connected by a nickel e-textile material (nickel plated Less EMF Shieldit Super Fabric from Less EMF, Latham NY, USA, with a resistivity of 0.07 Ohm/sq). This material is thin and flexible, which makes it a suitable option for wearable electronics development. Moreover, the right-hand-side copper endpoints of the ICs are connected to a basic dipole antenna (presented previously in He et al. [23], Johanna et al. [24], dimensions 2 cm × 10 cm) with a copper e-textile pattern (LessEMF pure copper polyester taffeta fabric, with a resistivity of 0.05 Ohm/sq). The dipole antenna is cut from the nickel e-textile.

The top layer is also constructed of the nickel e-textile, which is attached on the cotton-based fabric. As shown in Figure 1b, the shape of the electro-textile pattern is designed to match the left-hand-side copper endpoints of the ICs on the bottom layer. In addition, an elastic and flexible cell rubber (EPDM) material is used as the isolation layer to separate the top and bottom layers. All three layers are finally matched carefully and then fixed together by embroidering.

**Figure 2:**
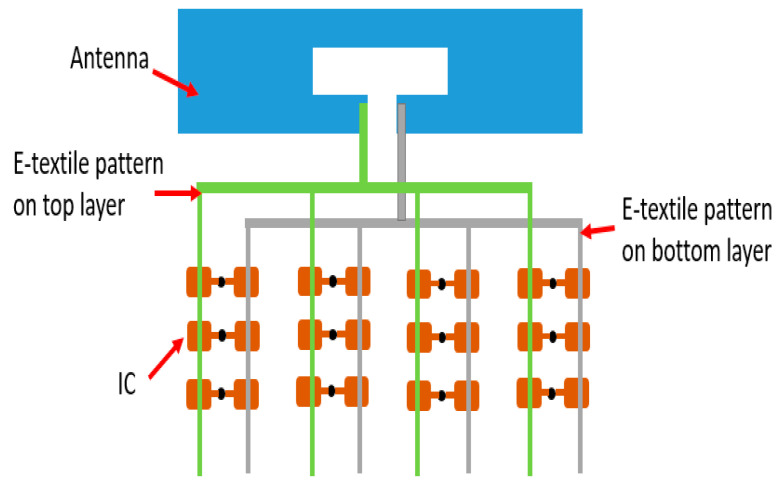
Illustration of the platform design.

**Figure 3:**
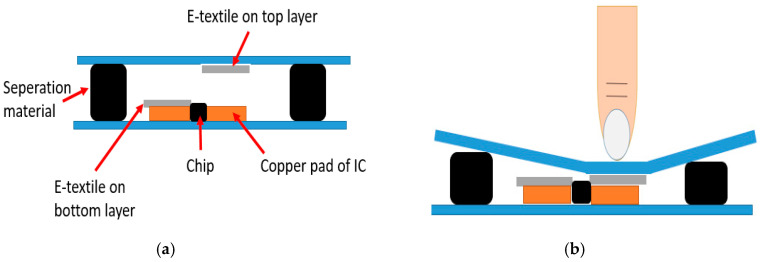
Status of the platform. (**a**) Before touching; (**b**) during touching.

To better understand the connections of RFID ICs, the design of the platform is illustrated in Figure 2. The gray lines represent the e-textile pattern on the bottom layer, which cascades on one side of the ICs to the dipole antenna. The other sides of the ICs are connected by green lines that indicate the e-textile pattern on the top layer. Initially, the bottom and the top layer are isolated by the separation material. Thus, only one of the endpoints of the ICs on the bottom layer are connected to the antenna. Thus, none of the ICs is initially readable for the external RFID reader. This off-status is shown in Figure 3. When the surface of the textile platform is touched by a finger, the touch will create an electrical connection between the top and bottom layers. Due to this, the other endpoint of the IC is connected to the antenna through the e-textile connectors on the top layer. Subsequently, the touched ICs are detected by the RFID reader. Thus, the finger touch location is determined according to the recorded IC’s unique ID.

**Figure 4:**
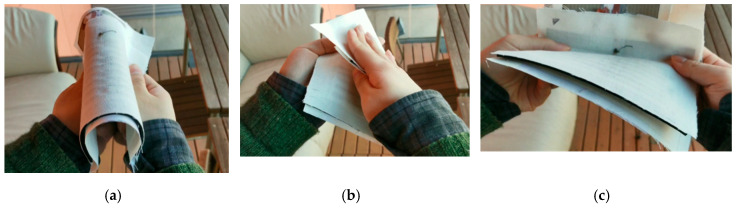
Initial stability test of the integrated circuits (IC) platform before writing. (**a**) Bent; (**b**) folded; (**c**) curved.

As shown in Figure 4, before starting to write on the platform, the platform was bent, folded and curved a few times (3–5), to ensure its firm structure. This type of mechanical stresses did not harm the platform performance, which can be considered promising when thinking about the future long-term reliability evaluations.

## 3. Character Recognition

Both spatial and sequential information are received from the device. The ICs are named based on their row and column coordinates, both ranging between 1 and 10. Thus, each of the 100 chips have a unique ID and return a temporal sequence for our character recognition. Using this data, a 10 × 10 bitmap can be formed. On the other hand, also the order of the activated ICs is known, so one can format the data as a sequence ordered by time, as well.

We use the platform for character recognition and experiment on the resulting accuracy in a live setup. The recognition algorithm is trained off-line using a separately collected training dataset for this task. The training dataset consists of 4500 manually labeled handwritten digits from six individual test subjects, representing five different nationalities. Each subject collected 75 samples of each digit from 0 to 9, totaling 750 digits per subject. We intentionally collected a new dataset instead of using an existing one, since the goal was to get the character recognition working on the device as well as possible, and the data differed quite a lot from traditional character recognition tasks. MNIST (Modified National Institute of Standards and Technology) database could probably be used to pretrain the network, but one would still need to fine-tune it with our data afterwards. The raw data were then formatted to 10 × 10 bitmaps and a time series of 100-dimensional-one-hot-encoded vectors. The sequences were limited to 100 time-steps since the sequences were rarely longer than that.

**Figure 5:**
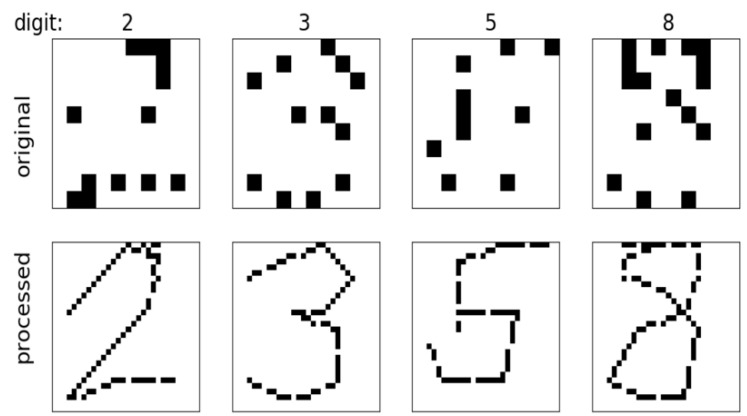
Samples from the dataset. (**top**) raw data; (**bottom**) interpolated data.

There are some errors in the raw data: After too many times of touching and pressing, the shape of the surface of the platform changed slightly, which led to slight misalignment problem. Thus, some undesired ICs were activated during the data collection. Moreover, there were some missed IC activations due to the obstruction and interferences from the environment. However, the effect of these errors could be eliminated by processing the data properly. Since the order of IC activations is known, one can interpret the order of the touch stroke in addition to the digit shape. The stroke order can then be used to interpolate between neighboring samples, increasing classification accuracy. It was also found, that by increasing the bitmap resolution to 30 × 30, the accuracy increased as well [25]. Examples of unprocessed bitmaps are presented in the top row of Figure 5, while the same samples after preprocessing are presented in the bottom row. The interpolation makes figures and the digits in them much clearer, as shown in Figure 5. However, the final 30- × 30-pixel image is the static image of the completed image and it is the one used as input for image recognition with CNN (convolutional neural network).

**Figure 6:**
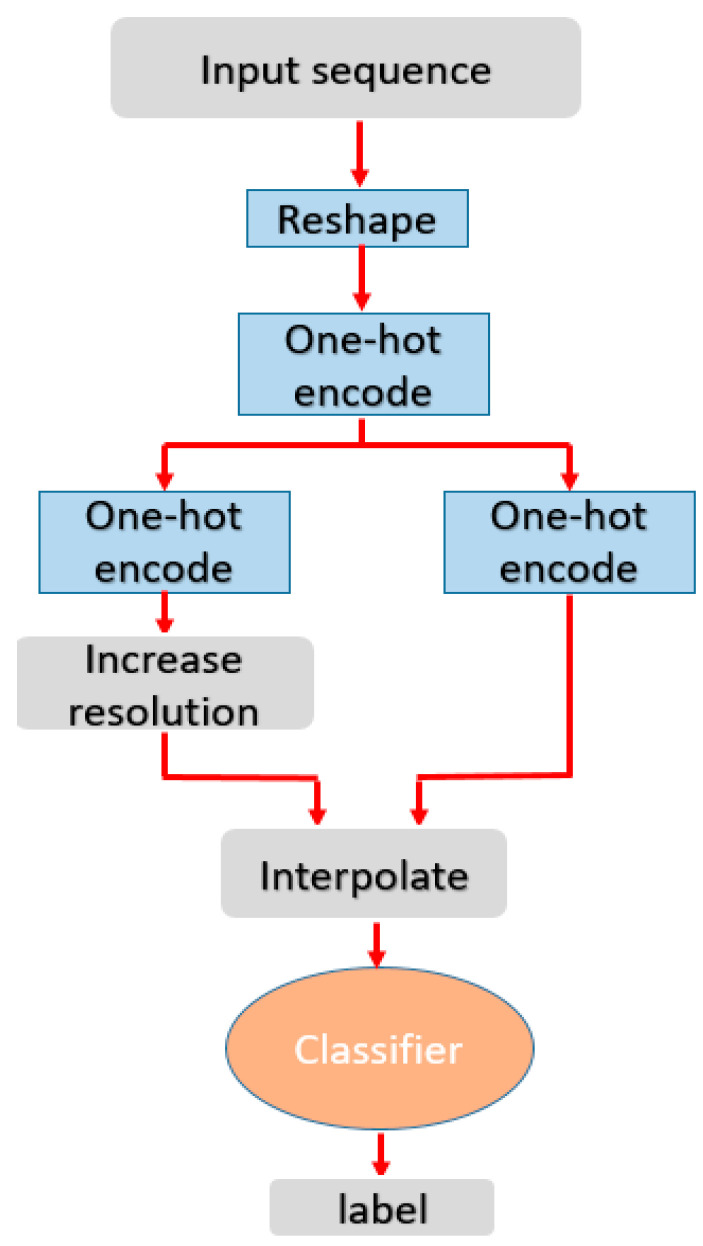
Character recognition pipeline.

A CNN was trained with the handwritten digit dataset. Since the bitmap resolution was increased to 30 × 30, a deeper network architecture, with multiple convolution layers and max-pooling, could be used. The deeper architecture seems to distinguish digits better than a shallower one using 10 × 10 input [25]. While the classifier is relatively deep for handwritten character recognition, with roughly 86,000 trainable parameters, it is still rather small and can easily be used for real-time recognition, even on mobile devices. Due to the errors mentioned before, the deeper structure is found to be necessary, as it increases the translational invariance due to a larger receptive field. The entire classification pipeline is depicted in Figure 6.

The fully convolutional architecture is as follows: First, two convolution blocks followed by a max pooling layer are repeated twice. This is followed by one more convolution block, global average pooling, fully connected layer and a softmax activation layer. The convolution blocks are identical save hyperparameters: a convolutional layer, a batch normalization layer, ReLU activation and a dropout layer. The convolutional layers of the first two blocks have 32 filters and kernel size of 4 × 4 pixels. Last three have 64 filters and 2 × 2 kernels. This is the smallest architecture presented in [25], chosen since it has the smallest computational footprint and is thus most suitable for embedded systems with limited resources, e.g., wearables. The deeper architectures in [25] could also be applied if the accuracy of the one used is determined to be too low.

## 4. Real-Time Recognition and Results

**Figure 7:**
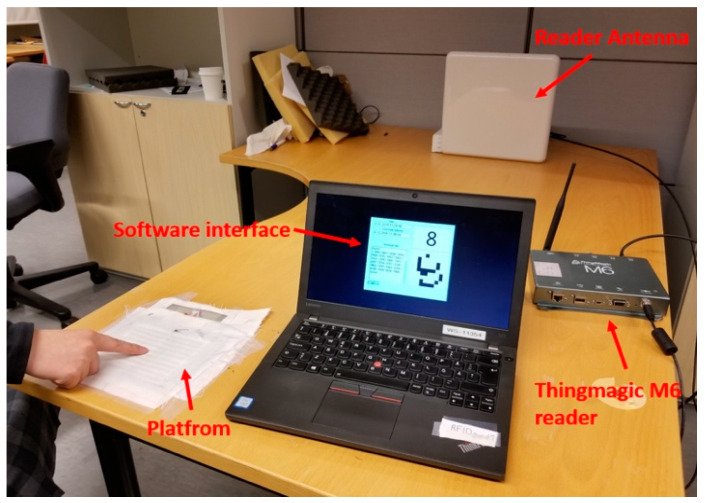
Real-time testing in a normal office environment.

Figure 7 shows the measurement setup that includes the ClothFace textile platform, a circularly polarized reader antenna, attached to Thingmagic M6 RFID reader through a connecting cable and our testing software user interface. The reader operates at the European standard frequency range (865.6–867.6 MHz) and the used power is 28 dBm. All the testing was implemented in a working office, under the effects of the surrounding environment, including computers, Wi-Fi and mobile phone signals, people and office furniture.

Two women and four men with various nationalities participated in the first real-time testing. Two of the testers were involved in collecting the training datasets for the character recognition, which also means that they are familiar with the platform. The rest of the testers have not used the textile platform before. This way it was possible to test if being part of training could improve the user’s character recognition. Every tester wrote each digit (0–9) 30 times during the testing. Thus, 1800 of handwritten digits in total was completed by all the testers to evaluate the real-time performance of the platform. In this first testing data from six subjects the erroneous classifications were not recorded, i.e., to what characters the erroneously classified characters were classified. To have data on this, a second real-time testing was done, in which two testers—a man and a woman—wrote each digit 50 times. Thus, 1000 of handwritten digits in total was completed by the two testers.

**Figure 8:**
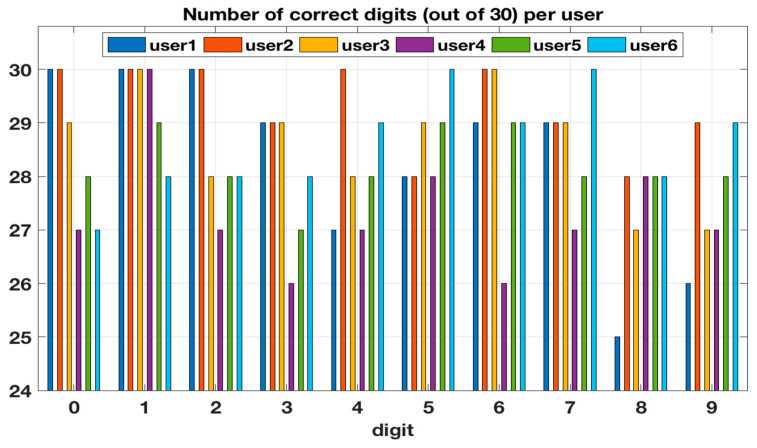
Number of correct digits for each user.

**Figure 9:**
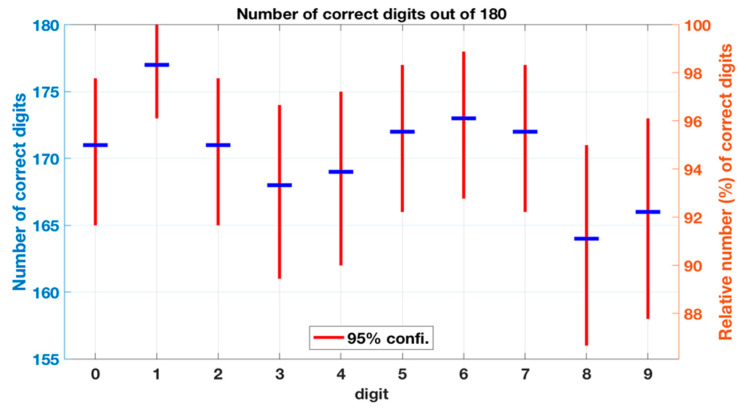
Number of correctly classified inputs for each digit with the confidence intervals for each digit.

The overall success rates of the real-time digital inputs for each user and in total from the first test with six participants are shown in Figure 8 and Figure 9, respectively. The results from the second test with two participants are shown in Table 1 as a confusion matrix. Figure 9 also shows the 95% confidence intervals for each digit. The confidence intervals were estimated with the bootstrapping method: The testing data were randomly sampled with replacement ten thousand times. Each sampling generated data similar to the original data, i.e., a set of 180 classification results for a digit. Next, the number of correctly classified digits for each sample was calculated and these values were sorted from the lowest to the largest. Then the 250th and 9750th values define the 95% confidence intervals.

**Figure 10:**
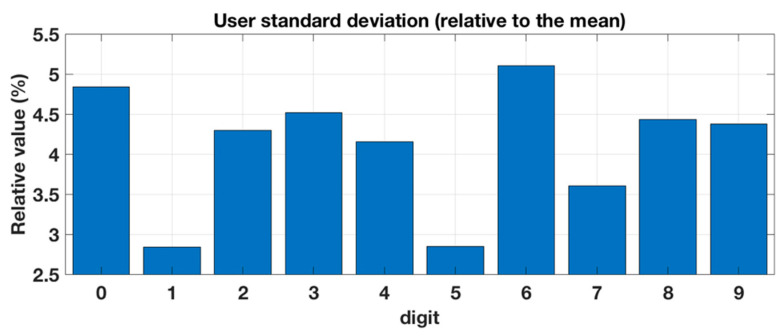
Standard deviation (relative to the mean) between the users for each digit.

In the first test with six participants the overall recognition accuracy of the platform was 94.6% (91.2–97.6) and the accuracy for each digit was between 91.1% (86.7–95.0) and 98.3% (96.1–100.0). Digit 8 had the lowest correct rate, while Digit 1 had the highest. Two users, user 2 and 3, were part of both training and testing. Their mean recognition accuracy was 96.5% (95.0–97.8) whereas for the other four users the mean recognition accuracy was 93.7% (92.3–95.0). This difference was statistically significant (*p* = 0.003). Figure 10 shows the standard deviation (relative to the mean) between the users for each digit in the first testing. As can be seen, Digits 0 and 6 had the most variance between the users, while for Digits 1 and 5 the platform had the most stable recognition performance.

In the second test with two participants the overall recognition accuracy was 96.8% (95.4–97.7). All the classification result of the second test are shown in the confusion matrix in Table 1. The overall precision and recall were 0.968 which means that the overall F1 score was 0.968. The precision recall and F1 score for each digit were also shown in Table 1 and Digits 0 and 1 had the highest F1 scores whereas Digit 3 had the lowest F1 score. Digit 3 had the lowest precision whereas Digit 8 had the lowest recall.

## 5. Discussion

ClothFace platform is not only simple and reliable, providing high accuracy, but also extremely flexible, allowing each user to use their personal handwriting. The goal of this study was to introduce this new type of multiple IC platform at a proof-of-concept level. Thus, no reliability evaluation in a real use environment (e.g., when integrated into clothing and worn on body) was done yet. However, before any testing, the platform was folded and curved by hand, and according to the achieved results, it still works well after these mechanical stresses. More, during the data training, 4500 handwritten digits were collected by one platform and after this, the platform still showed great performance in real-time testing. Further, the used materials and manufacturing methods (e.g., electro-textiles and embroidery) were previously used in textile electronics structures that showed excellent reliability under mechanical stresses [26,27].

The handwriting recognition platform in real-time use was tested in two tests, the first with six and the second with two participants, and the correct recognition rates was about 95%. In the second test the overall precision, recall and F1 score were 96.8%. In the first test, two users were also part of the training and their mean recognition accuracy was 96.5% which was statistically significantly higher than the mean recognition accuracy 93.7% of the other four users that did not participate in training. Thus, this indicates that being part of training can improve the user’s character recognition accuracy.

In the first test Digit 8 was the worst case and it could be recognized at least with 86.7% success rate with a 95% confidence. In the second test, the Digit 8 also had the lowest recall, while Digit 3 had the lowest precision. In both tests, Digit 1 had the highest recognition accuracy. From the confusion matrix, it can be seen also that the few errors were mostly what would be expected if a few pixels were missing: for example, Digits 3 and 5, 3 and 8, 7 and 9, 4 and 9 were the most often confused ones. The variability between the users was low, as there was a maximum of 13.3% (26 vs. 30 out of 30 for Digit 6) difference in the success rates between the users for a single digit.

These are very good numbers considering this is one of the first prototypes. It is easy to see the possibilities of improvement in the machine learning methods and in using more and different sources of training data. For example, CNNs of various implementations have had test error rates of 0.23–1.7 [28], which is better than the achieved about 5%. The more complex architectures in Raivio et al. [25] could increase the live accuracy even further in applications where robustness is important and thus may be tested in future works. There are also many things that can be improved in the design, material choices and manufacturing, for example by optimizing the antenna design for a specific use environment, selecting the most conductive electro-textiles and using an optimized number of ICs.

This technology enables our everyday clothing—as well as textiles around us—to transform into intelligent user interfaces. As the solution is passive, no on-board energy sources are needed. Thus, there is no need for maintenance. Possible users of ClothFace platform are especially special needs groups, such as elderly people and people with cognitive challenges. This platform is simple to use and, when integrated into clothing, always with the user. Whereas mobile devices require a certain amount of cognitive skills, they can be lost, or they may be out of the battery. As the technology can be powered by an external RFID reader and connected to any application through WIFI, it is possible to use the clothing-integrated interface for example in a care home or in a home environment. As the platform prototype is already fully textile-based, it shows that seamless integration of ClothFace technology into clothing is possible. However, it should be noted, that using the current type of platform on an irregular-shaped substrate may not be possible but would require use of different type of textile materials.

The next step is to optimize the platform to be used near the human body, for example when integrated to a shirtsleeve. The human body is lossy and moving, which causes challenges for on-body performance. Further, testing smaller antenna designs and reducing the size of the platform are important future steps: It will be easier to use the platform on body, when it requires a smaller flat surface. Further, another important step is to test the performance of the platform on a curved surface.

## 6. Conclusions

In this paper, we introduced a prototype of ClothFace technology, a passive UHF RFID-based handwriting recognition platform, integrated into a cotton fabric. The created, fully textile-based platform enables real-time number recognition. As the platform is functional without on-board energy sources, it will turn our everyday clothing and textiles around us into passive user interfaces. The handwriting recognition platform in real-time use was tested in two tests, the first with six and the second with two participants, and the correct recognition rates was about 95% in a normal office environment. It could be seen also that the few errors were mostly what would be expected if a few pixels were missing. However, our results indicate that being part of training can improve the user’s character recognition accuracy. These results are very encouraging—especially when considering that the handwriting recognition on a fully textile-based platform, being a simple, cost-effective and flexible solution, promises versatile application areas in numerous different contexts. Next, to expand the application of ClothFace platform, character recognition (A–Z) will be implemented. In addition, the design will be further developed by reducing the size and seamlessly integrating it into a shirtsleeve or into a pair of pants.

## Figures and Tables

**Table 1:** Confusion matrix for real-time recognition testing with metrics (precision, recall, F1 score) for each digit.

		Detected Digits										Metrics		
		0	1	2	3	4	5	6	7	8	9	Precision	Recall	F1 Score
**Written Digits**	**0**	100										1.00	1.00	1.00
	**1**		100									0.99	1.00	1.00
	**2**			96	3		1					1.00	0.96	0.98
	**3**				95		2			3		0.91	0.95	0.93
	**4**					96					4	1.00	0.96	0.98
	**5**				2		96	2				0.96	0.96	0.96
	**6**						1	99				0.96	0.99	0.98
	**7**		1						97		2	0.95	0.97	0.96
	**8**				4			2		94		0.97	0.94	0.95
	**9**								5		95	0.94	0.95	0.95
